# Investigating the Prevalence of Reactive Online Searching in the COVID-19 Pandemic: Infoveillance Study

**DOI:** 10.2196/19791

**Published:** 2020-10-27

**Authors:** Rafael A Badell-Grau, Jordan Patrick Cuff, Brendan P Kelly, Helen Waller-Evans, Emyr Lloyd-Evans

**Affiliations:** 1 School of Biosciences Cardiff University Cardiff United Kingdom; 2 Medicine Discovery Institute Cardiff University Cardiff United Kingdom

**Keywords:** chloroquine, coronavirus, COVID-19, fake news, Google Trends, ibuprofen, infodemiology, misinformation

## Abstract

**Background:**

The ongoing pandemic has placed an unprecedented strain on global society, health care, governments, and mass media. Public dissemination of government policies, medical interventions, and misinformation has been remarkably rapid and largely unregulated during the COVID-19 pandemic, resulting in increased misinterpretations, miscommunication, and public panic. Being the first full-scale global pandemic of the digital age, COVID-19 has presented novel challenges pertinent to government advice, the spread of news and misinformation, and the trade-off between the accessibility of science and the premature public use of unproven medical interventions.

**Objective:**

This study aims to assess the use of internet search terms relating to COVID-19 information and misinformation during the global pandemic, identify which were most used in six affected countries, investigate any temporal trends and the likely propagators of key search terms, and determine any correlation between the *per capita* cases and deaths with the adoption of these search terms in each of the six countries.

**Methods:**

This study uses relative search volume data extracted from Google Trends for search terms linked to the COVID-19 pandemic alongside *per capita* case and mortality data extracted from the European Open Data Portal to identify the temporal dynamics of the spread of news and misinformation during the global pandemic in six affected countries (Australia, Germany, Italy, Spain, the United Kingdom, and the United States). A correlation analysis was carried out to ascertain any correlation between the temporal trends of search term use and the rise of *per capita* mortality and disease cases.

**Results:**

Of the selected search terms, most were searched immediately following promotion by governments, public figures, or viral circulation of information, but also in relation to the publication of scientific resources, which were sometimes misinterpreted before further dissemination. Strong correlations were identified between the volume of these COVID-19–related search terms (overall mean Spearman rho 0.753, SD 0.158), and *per capita* mortality (mean *per capita* deaths Spearman rho 0.690, SD 0.168) and cases (mean *per capita* cases Spearman rho 0.800, SD 0.112).

**Conclusions:**

These findings illustrate the increased rate and volume of the public consumption of novel information during a global health care crisis. The positive correlation between mortality and online searching, particularly in countries with lower COVID-19 testing rates, may demonstrate the imperative to safeguard official communications and dispel misinformation in these countries. Online news, government briefings, and social media provide a powerful tool for the dissemination of important information to the public during pandemics, but their misuse and the presentation of misrepresented medical information should be monitored, minimized, and addressed to safeguard public safety. Ultimately, governments, public health authorities, and scientists have a moral imperative to safeguard the truth and maintain an accessible discourse with the public to limit fear.

## Introduction

The COVID-19 pandemic has encouraged an unprecedented international panic. Since its emergence in late 2019 in the Hubei Province of China, COVID-19 has spread worldwide, and its associated infectivity and death rate have challenged world leaders, health care systems, and the public [1,2]. Unlike comparable previous pandemics, such as the Spanish flu in 1918, the internet has provided to the public a source of connectivity and a means to rapidly acquire emerging information about the virus [1]. The information available is, however, not always verifiable or scientifically supported.

The dissemination of government policy and cutting-edge medical research is unquestionably important in the remit of a global pandemic, but misinterpretation is commonplace. The desperation of the public encourages the opportunistic adoption of unverified medical interventions. The misuse and misrepresentation of such information presents a critical challenge to governments and to the public. Equally, the public may seek out and enable misinformation (eg, the virus being spread by 5G towers [3]), which is rapidly distributed via social media [4]. The increased dependence of the public on social media and other inherently biased sources of information may inflate the rate at which misinformation spreads, possibly fostering disenfranchisement with government and health care organizations [5-7]. This could ultimately provoke disregard toward restrictions enforced for public safety, lead to reduced supplies of medicines and personal protective equipment (PPE), or potentially even to reduced medical engagement and worsening of chronic conditions, increasing pressure on already strained health care providers.

Given the rapid flow of digital information during the COVID-19 pandemic, real-time data collection and analysis provides an unparalleled opportunity to assess the public response to information as it emerges. Through internet-derived information from social media, news, and search engine use, public reactions and perceptions can be assessed in real time [4,8-11]. Google Trends (GT) has been used for not only the analysis of epidemiologically relevant data regarding influenza [9] and disease outbreaks [10] but also, more recently, COVID-19 [11]. By assessing the temporal dynamics of search terms related to the pandemic, particularly those relating to misinformation, it is possible to infer likely sources, propagators, and impacts. This study employs GT for the analysis of search terms used during the COVID-19 pandemic relating to government policy, potential treatments, and misinformation, specifically in three English and three non-English speaking countries: Australia, the United Kingdom, the United States, Germany, Italy, and Spain. The aims of this study are to identify any correlation between the relative search volumes (RSVs) for information relating to the first wave of the pandemic, and to discuss these search volumes in the context of emerging news, alongside the prevalence of cases and deaths in each of the six focal countries.

## Methods

### Mortality, Case, and Testing Data Extraction

**Figure 1:**
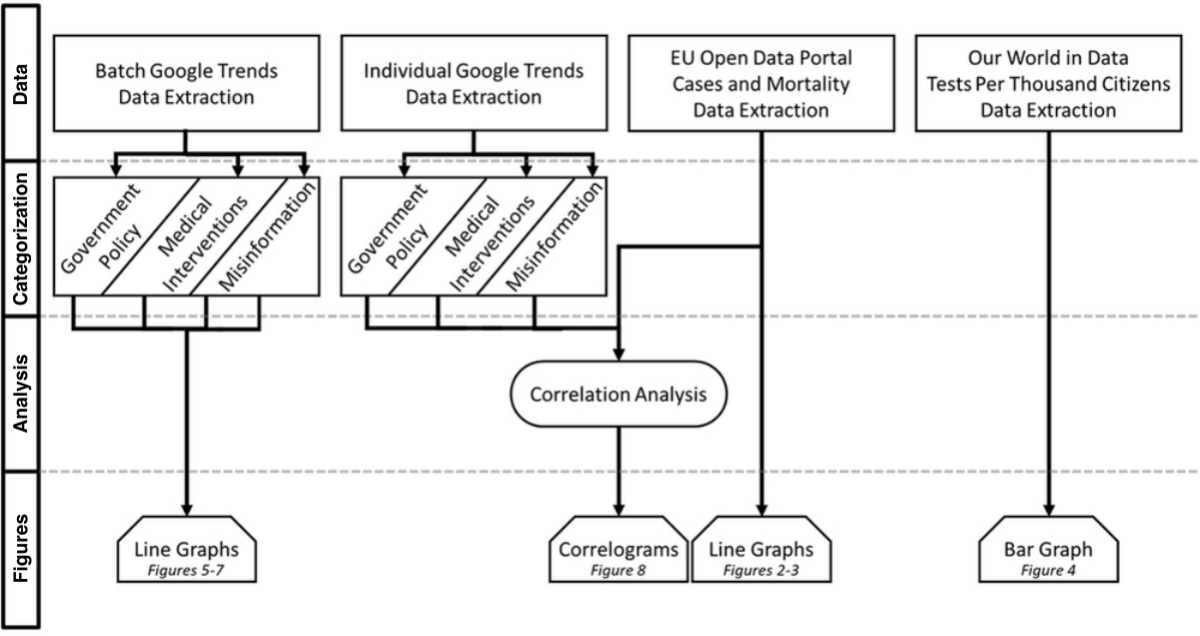
Data extraction and workflow.

Worldwide mortality and case data, and country population sizes were extracted from the European Union Open Data Portal [12] on April 17, 2020 (Figure 1). Data were retained only for the six focal countries. Dates for which no data were available from November 1, 2019, to the first recorded numbers for that country were marked as zero. *Per capita* cases and deaths were also calculated using the included population sizes and retained for later analyses and figures. *Per capita* values, although not widely reported by the media at this time, were used in this study to correct for the large variation in population sizes of the focal countries, and to better represent the proportional pressure upon each country.

The objective reliability of these data is questionable given the internationally variable extent of testing and the resultant predicted inaccuracy of the case numbers in each country. International variations in the definition of COVID-19–related deaths and failures to report the full extent of case numbers also warrant skepticism. In the remit of this study, however, these data represent the immediate perceived threat and pressure elicited upon the societies of each focal country, thus providing a suitable comparison against the temporal dynamics of the search terms used. The numbers of COVID-19 tests per thousand citizens were downloaded from Our World in Data [13]; given the irregularity of testing and resultant unavailability of data for some countries, these data were not used for correlation analysis. The number of tests completed by April 17, 2020, was recorded, except for Germany and Spain for which values represented the tests per thousand completed by April 19 and 13, respectively, due to a lack of data for April 17. Testing data represent the number of tests performed, rather than the number of individuals tested, given the wider availability of these data; the nature of Australia’s testing units is, however, unclear.

### Search Volume Data Extraction

Data were extracted from GT on April 17, 2020, for the period of November 1, 2019, to April 17, 2020, which includes a brief period before the first confirmed case of COVID-19 for comparison. These data provide a proxy for public interest in government policy, emerging health care interventions, and misinformation, later contextualized as a response to the release of such information. The data extracted from GT are RSVs for predetermined search terms, allowing comparison of search rates for different terms via Google, the most widely used internet search engine, especially in the countries selected [14,15]. These RSVs are presented for each date of a given time period within a given country. Data are normalized relative to the highest RSV peak in that time period (this peak represented as 100).

Data were extracted for searches generated from Australia, Germany, Italy, Spain, the United Kingdom, and the United States. These countries were selected due to their widespread use of Google (precluding China and many other Asiatic countries), variation in the extent to which they were impacted by the pandemic, nuances in their responses to the pandemic, and the accessibility of their news and media in one predominant language. All search terms were preceded by “coronavirus” to ensure relevance to the pandemic; “coronavirus” was selected over “COVID19” and similar terms due to its greater prevalence of searches (eg, in the United States, “coronavirus vaccine” yielded four-fold the search volume of “corona vaccine” and “covid vaccine”, and twenty-fold that of “covid19 vaccine” and “covid-19 vaccine”).

All search terms were selected based on their widespread media coverage and their high Google search volumes. Their placement in the broad categories of *government policy*, *medical interventions*, and *misinformation* were based on the context of their wide reporting by media, government, research, and health care organizations of those particular countries. The designation of search terms as *medical interventions* did not equate to their effectiveness in treating COVID-19 but scientific discussion around, or political endorsement of, their experimental or genuine use in treating the virus. Chloroquine, for example, was not empirically shown to benefit patients at the time of this study, and its early endorsement during the pandemic largely emanated from the United States, but international research nonetheless endeavored to ascertain any benefit it conferred to patients with COVID-19, this being the primary focus of its initial widespread news coverage. Misinformation search terms were labeled as such when there was no empirical evidence nor active published peer-reviewed research regarding their relevance to COVID-19, and their media coverage indicative of their potential for controversy; such search terms could often be traced back to an initial misinterpretation or false statement, some of which are highlighted in the discussion. All terms were identified as COVID-19 misinformation by Dhillon et al [16]. Other search terms relevant to COVID-19 were considered but for a contained and meaningful statistically significant comparison only those with relatively high and comparable RSVs within the three aforementioned categories were included. Search terms for which variations were possible (eg, chloroquine vs hydroxychloroquine) were included as the variation with the greatest GT search volume with the simpler terminology routinely having the greatest search volume.

Searches were carried out in the language native to each country unless the English terms provided a greater number of results (ie, where English phraseology was adopted). Searches were carried out in batches to identify relative differences in search volumes, with three batches coarsely defined as “government policies,” “medical interventions,” and “misinformation.” All search batches contained “coronavirus chloroquine” as a standard to facilitate some comparison between categories given its relatively central positioning in most batches. Chloroquine was selected for its relatively average search volume across countries and categories, acting as an anchor to facilitate visual comparison between higher and lower RSVs. The search term RSVs were all also individually downloaded (independently normalized with the highest peak being 100) for subsequent correlation analysis to evenly represent the extent of searching and focus on the temporal dynamics. Given the representation of numbers less than one as “<1” by GT, all RSVs of “<1” were converted to 0.5 to facilitate quantitative comparison.

The government policy search terms comprised chloroquine (control standardization term), social distancing, sanitizer, mask, isolation, gloves, and testing (Textbox 1). Social distancing was implemented by many countries as an early and maintained means to prevent viral spread, as was isolation, although the latter may also have been searched in association with the well-being and mental health consequences of reduced social contact during lockdown. The use of sanitizer for cleansing of hands was also encouraged by governments throughout the pandemic, although depleting public availability in most countries led many to attempt to create homemade sanitizer [17]. Masks and gloves were employed as a protective means to prevent spread, although predominantly by frontline health care workers; public purchase of this PPE was problematic in many countries, resulting in reduced availability for medical practitioners [18,19]. Testing and tracing was carried out for coronavirus, but the extent of testing and the national focus on its importance varied internationally [13]. The US spelling of “sanitizer” was maintained for the UK searches given a higher prevalence than the UK spelling “sanitiser.” Due to the GT search limit, the government policy search was split into two batches (batch 1: chloroquine, social distancing, sanitizer, mask, and isolation, and batch 2: chloroquine, gloves, and testing, with linguistic variations for Germany, Italy, and Spain).

**Google Trends search terms used in each of the three categories.**

**Government policy**

- Australia, the United Kingdom, and the United States: social distancing, sanitizer, mask, isolation, gloves, testing
- Germany: social distancing, desinfektion-smittel, maske, isolation, handschuhe, testen
- Italy: distanziamento sociale, disinfettante, maschera, isolamento, guanti, analisi
- Spain: distanciamiento social, desinfectante, mascara, aislamiento, guantes, pruebas

**Medical interventions**

- Australia, the United Kingdom, and the United States: chloroquine, remdesivir, paracetamol, vaccine, ibuprofen
- Germany: chloroquin, remdesivir, paracetamol, impstoff, ibuprofen
- Italy: chlorochina, remdesivir, paracetamolo, vaccino, ibuprofene
- Spain: cloroquina, remedsivir, paracetamol, vacuna, ibuprofeno

**Misinformation**

- Australia, the United Kingdom, and the United States: 5G, man made, lab
- Germany: 5G, hergestellt, labor
- Italy: 5G, creato, laboratorio
- Spain: 5G, creado, laboratorio

The medical intervention search terms comprised chloroquine (control standardization term), remdesivir, paracetamol, vaccine, and ibuprofen (Textbox 1). All of these search terms pertain to treatments that were suggested to have potential effects against COVID-19 symptoms. The public focus on vaccines reflected the ongoing development of vaccines and the desire for relief from the pandemic [20]. Paracetamol and ibuprofen were used to subdue pain associated with COVID-19 symptoms, but public perception became antagonistic toward using ibuprofen for COVID-19 symptoms, which shifted focus toward paracetamol [21].

The misinformation search terms comprised chloroquine (control term), 5G, man made, and lab (Textbox 1). These search terms pertain to internationally prevalent misinformation related to COVID-19, often specifically suggesting a disingenuous cause or source of the viral spread. Specifically, these entail theories that the virus was being spread by the new 5G phone masts, that the virus was manufactured, and that the virus was released from a laboratory [3,16,22-24], all of which have subsequently been debunked [25-27].

### Statistical Analysis

Statistical analyses and plotting of data were carried out using R version v4.0.0 (R Foundation for Statistical Computing) [28]. Line graphs were created for *per capita* cases and deaths, and a bar chat for tests per thousand citizens using *ggplot* in the *ggplot2* package version 3.3.0 in R [29], with colors assigned via the *RColorBrewer* package v1.1-2 [30]. The data were identified as nonnormally distributed via Shapiro–Wilk tests, so nonparametric statistical analyses were selected. Correlations between RSVs and *per capita* deaths and cases were tested using Spearman rho rank correlation via the *rcor* function of the *Hmisc package* version 4.4-0 [31]. The output was then presented in a correlogram via the *corrplot* function of the *corrplot* package version 0.84 [32], with colors assigned via the *viridis* package v0.5.1 [33]. Line graphs were created for each of the three categories of search terms for each country to aid comparison of both the extent and temporality of RSV trends in GraphPad Prism version 8 (GraphPad Software) [34]. All statistical data is included in Multimedia Appendix 1.

### Information Sources and Reliability

The sources for the non–search term data (The European Union Open Data Portal and Our World in Data) are reputable sources that derive their data from official national reports, scientific publications, and other reliable sources. The data extracted from these sources align with those published internationally in response to the pandemic situation as it develops. The GT data are collected and presented by Google based on the input of users of their service, thus should be fully reliable. Although most sources cited in this report are from reputable scientific, government, or public health authority sources, others discussed throughout the manuscript are taken from mass media, social media, and other heavily biased sources or from scientific articles that discuss such sources; these sources are being referred to on the basis of these biases or simply to refer to the temporal development and emergence of global news, for which bias in an important factor. The paper discusses the reporting of this information in an objective manner, with no subscription to the reported ideals or beliefs represented in the text.

## Results

### Mortality, Case, and Test Results

**Figure 2:**
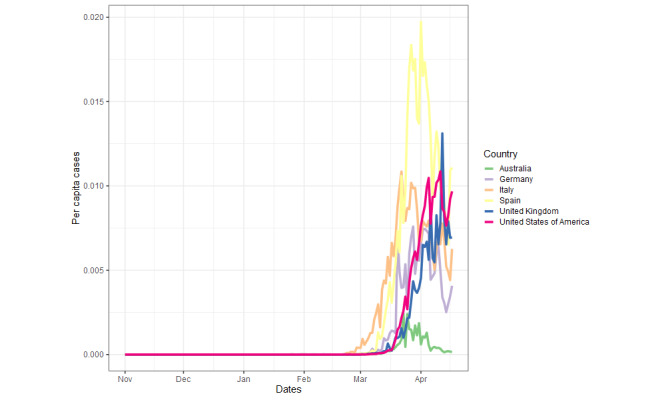
Per capita cases of COVID-19 during the study period.

**Figure 3:**
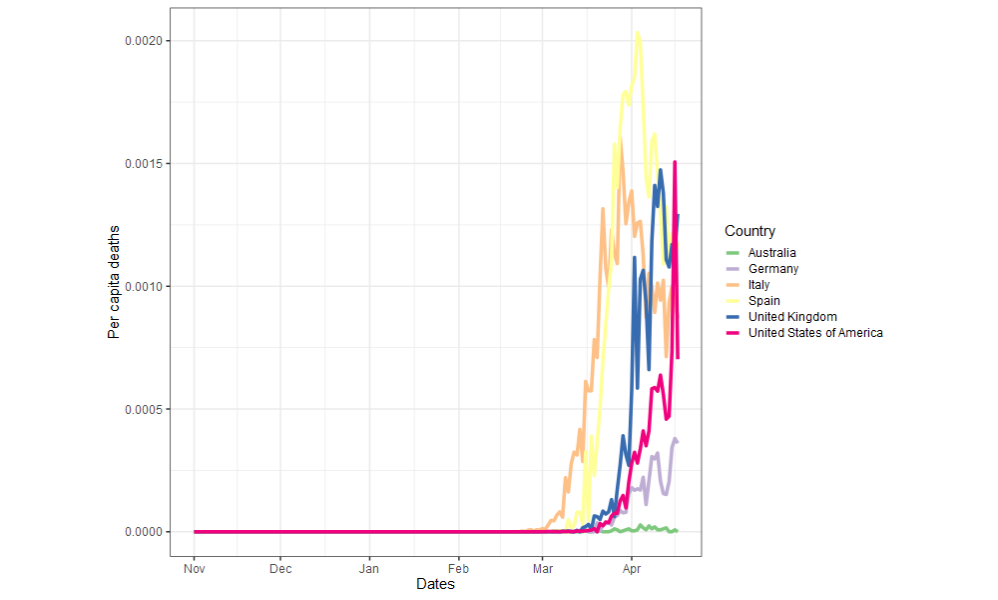
Per capita COVID-19–related deaths during the study period.

**Figure 4:**
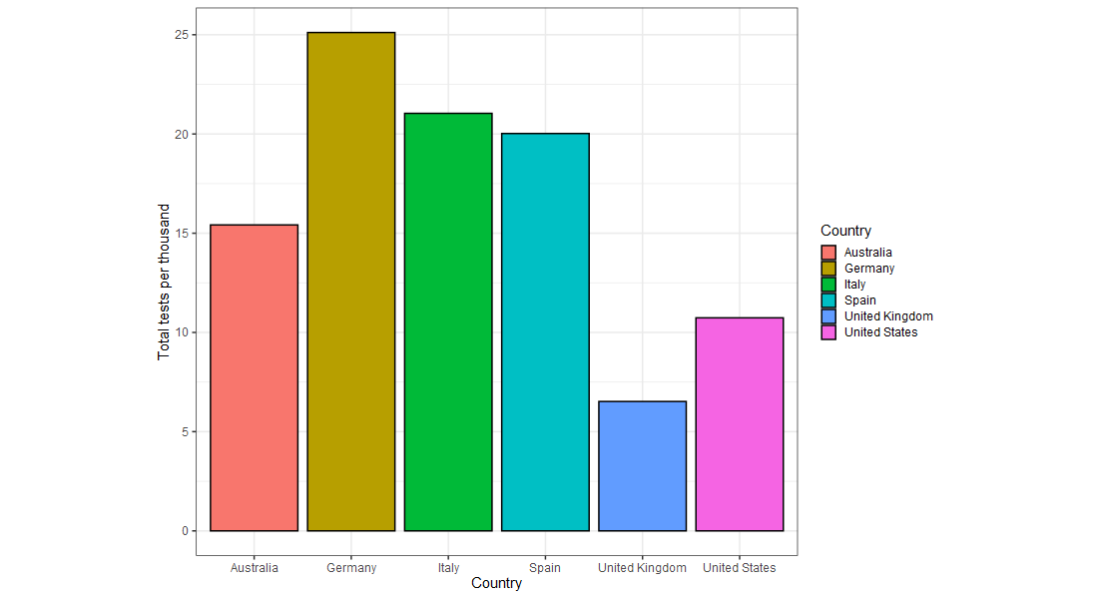
Total COVID-19 tests per thousand citizens in the six focal countries, as of April 17, 2020, except for Germany and Spain, which are represented by April 19 and 13, respectively, due to a lack of data for April 17.

All countries show similar *per capita* case (Figure 2) and death (Figure 3) trends temporally, with both beginning to exponentially increase in most countries between late February and early March. Of the six countries, Italy was the first to present a substantial number of cases and deaths (mid-February 2020). Australia, the United Kingdom, and the United States were the last to experience rapidly increasing *per capita* case numbers (~March 10). The *per capita* case number trends are relatively similar in their extents for most countries, except for Spain, which exhibits approximately 50% more peak *per capita* cases than the second highest peak, the United Kingdom (Spain: 0.01937, UK: 0.013113), and Australia, which exhibits approximately a quarter of the peak *per capita* cases for the majority of the countries (Australia: 0.002445, average for other countries, excluding Spain: 0.010603, SD 0.0023). The *per capita* deaths similarly increase last for Australia, the United Kingdom, the United States, and Germany. Germany and the United States display a shallower trajectory of *per capita* death increases, and Australia shows a minor peak of *per capita* deaths. Spain again exhibits the greatest peak of *per capita* deaths, but only with an approximate 30% increase over the peaks of Italy, the United Kingdom, and the United States (Spain: 0.002033, Italy: 0.001607, the United Kingdom: 0.001474, the United States: 0.001506), compared to the ~50% increase over the second highest peak for *per capita* cases. Testing for COVID-19 varied massively between countries, with Germany showing the highest tests per thousand, with around 25 tests per thousand, and the United Kingdom showing the lowest with around 6.5 tests per thousand (Figure 4).

### Search Volume Results

**Figure 5:**
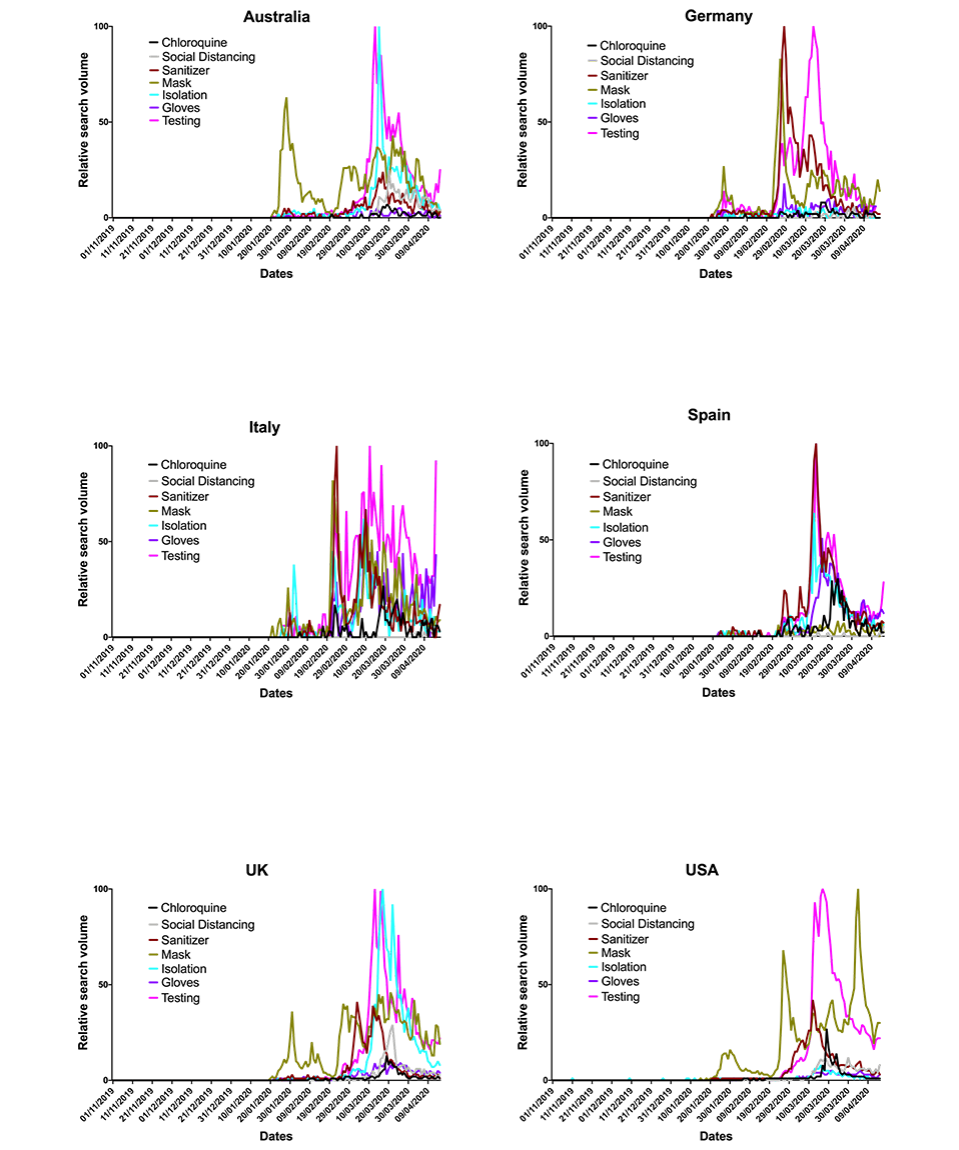
Government policy relative search volumes (RSVs) extracted from Google Trends (GT). Grouped RSV data, normalized to the highest RSV peak in the time period (represented as 100) were extracted from GT on April 17, 2020, for the period of November 1, 2019, to April 17, 2020. Search terms included “coronavirus chloroquine” (control term), “coronavirus social distancing,” “coronavirus sanitizer,” “coronavirus mask,” “coronavirus isolation,” “coronavirus gloves,” and “coronavirus testing,” with variations to reflect the language native to each country.

Of the government policy search terms (Figure 5), “testing” was prevalent in all countries, and “isolation” relatively high in all but the United States and Germany. “Sanitizer” was highly searched in Germany, Italy, and Spain. “Masks” was highly searched in Australia, Germany, Italy, and the United States, and to a lesser extent the United Kingdom. “Gloves” was searched less in all but Italy and Spain. “Social distancing” was searched less except in Australia and the United Kingdom, where this term was the third most searched. Most search terms peaked at a similar time (mid-March) in most countries, although “mask” also peaked in late January to early February in Australia, the United Kingdom, and the United States, and to a lesser extent in Germany and Italy. In Germany and Italy, “sanitizer” and “mask” peaked in early March, 2-3 weeks earlier than a later peak coinciding with that of “testing” in other countries. In the United States, “gloves” was searched most at the end of February, but also with a second peak in early April, unlike the other countries. In Italy, searches of “testing” peaked sporadically from late February to mid-April (the end of the search period), with larger peaks spread further across the period.

**Figure 6:**
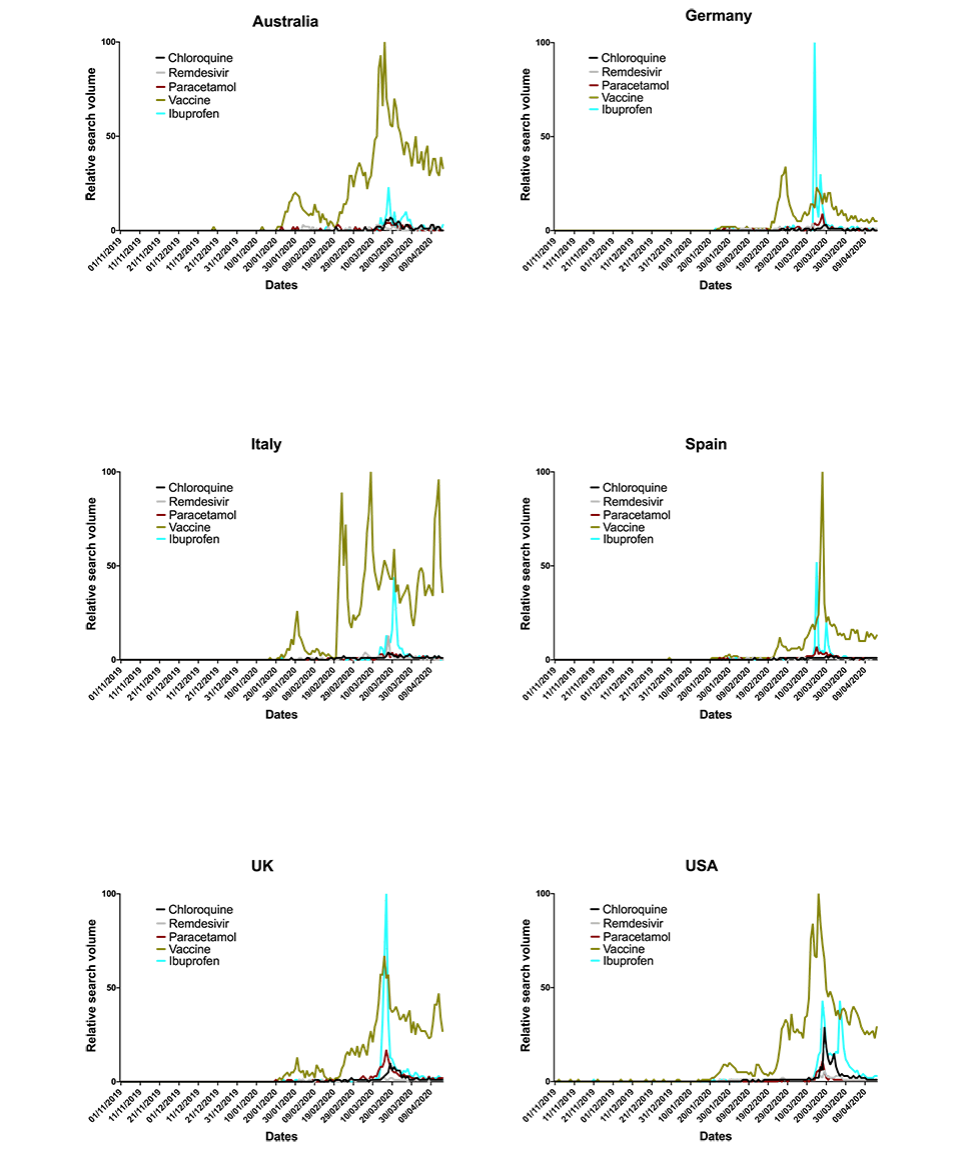
Medical intervention relative search volumes (RSVs) extracted from Google Trends (GT). Grouped RSV data, normalized to the highest RSV peak in the time period (represented as 100) were extracted from GT on April 17, 2020, for the period of November 1, 2019, to April 17, 2020. Search terms included “coronavirus chloroquine,” “coronavirus remdesivir,” “coronavirus paracetamol,” “coronavirus vaccine,” and “coronavirus ibuprofen,” with variations to reflect the language native to each country.

Of the medical intervention search terms (Figure 6), “vaccine” was highly searched in all countries, peaking in late March, except in Germany, where it peaked in late February, and Italy, where it peaked sporadically from the end of January to mid-April (the end of the search period). In the United Kingdom, “vaccine” had a second peak in mid-April. The other medical interventions had relatively small peaks, often in mid- or late March. “Remdesivir” peaked higher in Italy relative to the other countries. “Chloroquine” peaked much higher in the United States relative to the other countries, also having a smaller peak in the United Kingdom. “Ibuprofen” had the highest peak in Germany and the United Kingdom, peaking in all countries in mid- or late March and having a second peak in early April in the United States.

**Figure 7:**
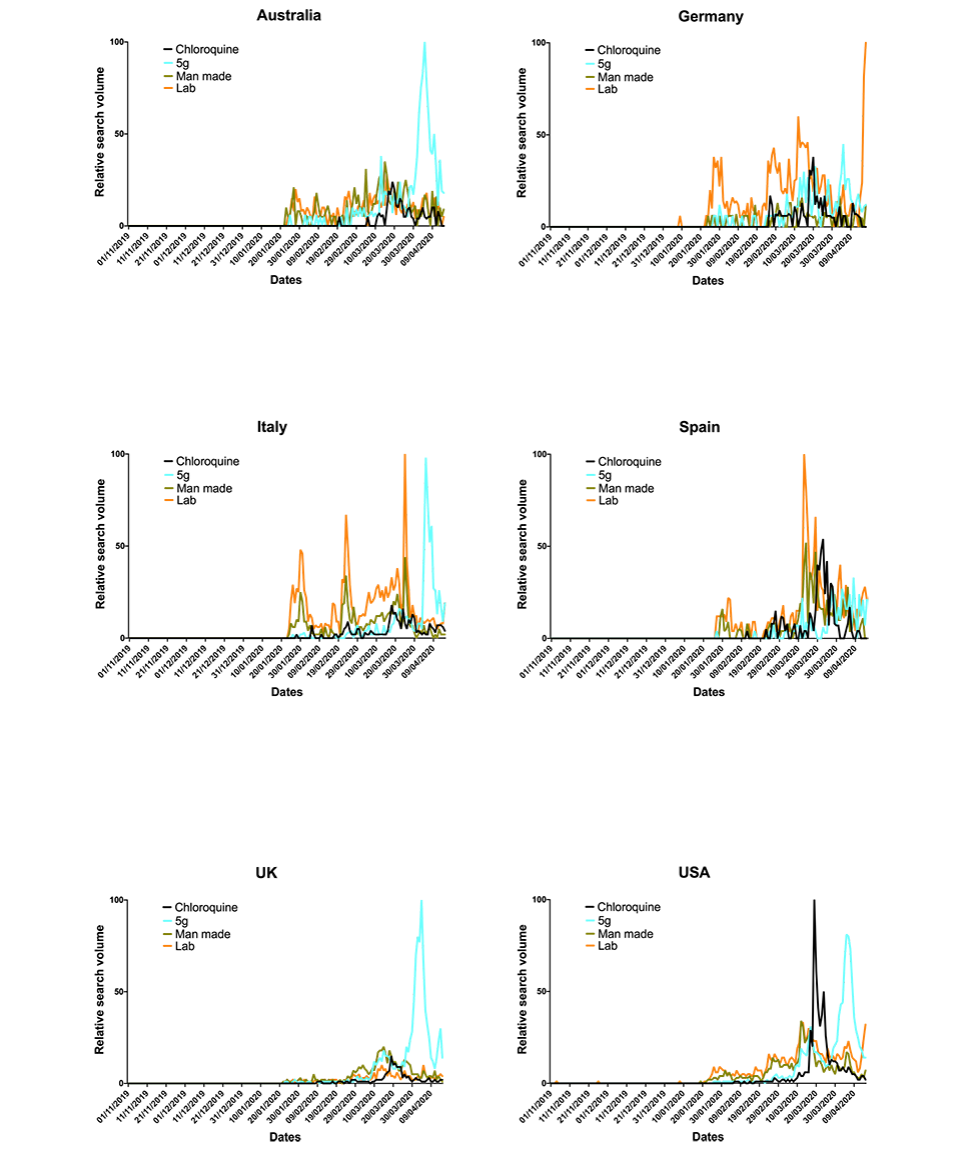
Misinformation relative search volumes (RSVs) extracted from Google Trends (GT). Grouped RSV data, normalized to the highest RSV peak in the time period (represented as 100) were extracted from GT on April 17, 2020, for the period of November 1, 2019, to April 17, 2020. Search terms included “coronavirus chloroquine,” “coronavirus remdesivir,” “coronavirus paracetamol,” “coronavirus vaccine,” and “coronavirus ibuprofen,” with variations to reflect the language native to each country.

Of the misinformation search terms (Figure 7), “5G” had erratic smaller peaks throughout mid- and late March but peaked in most countries in early April, with Germany and Spain displaying reduced peaks. “Man made” was mostly searched in mid-March, with some more widespread erratic peaks in all but the United Kingdom, and a substantial peak in late January and early February in Australia, Italy, and Spain. “Lab” was searched relatively little in Australia, the United Kingdom, and, to some extent, the United States. “Lab” was, however, highly searched in Italy and Spain in late March, with Italy also exhibiting large peaks in late January and late February, and was searched at similar intervals in Germany but never so proportionally high as Italy and Spain. In most cases, peaks of “lab” coincide with peaks of “man made.”

### Correlation Analysis Results

**Figure 8:**
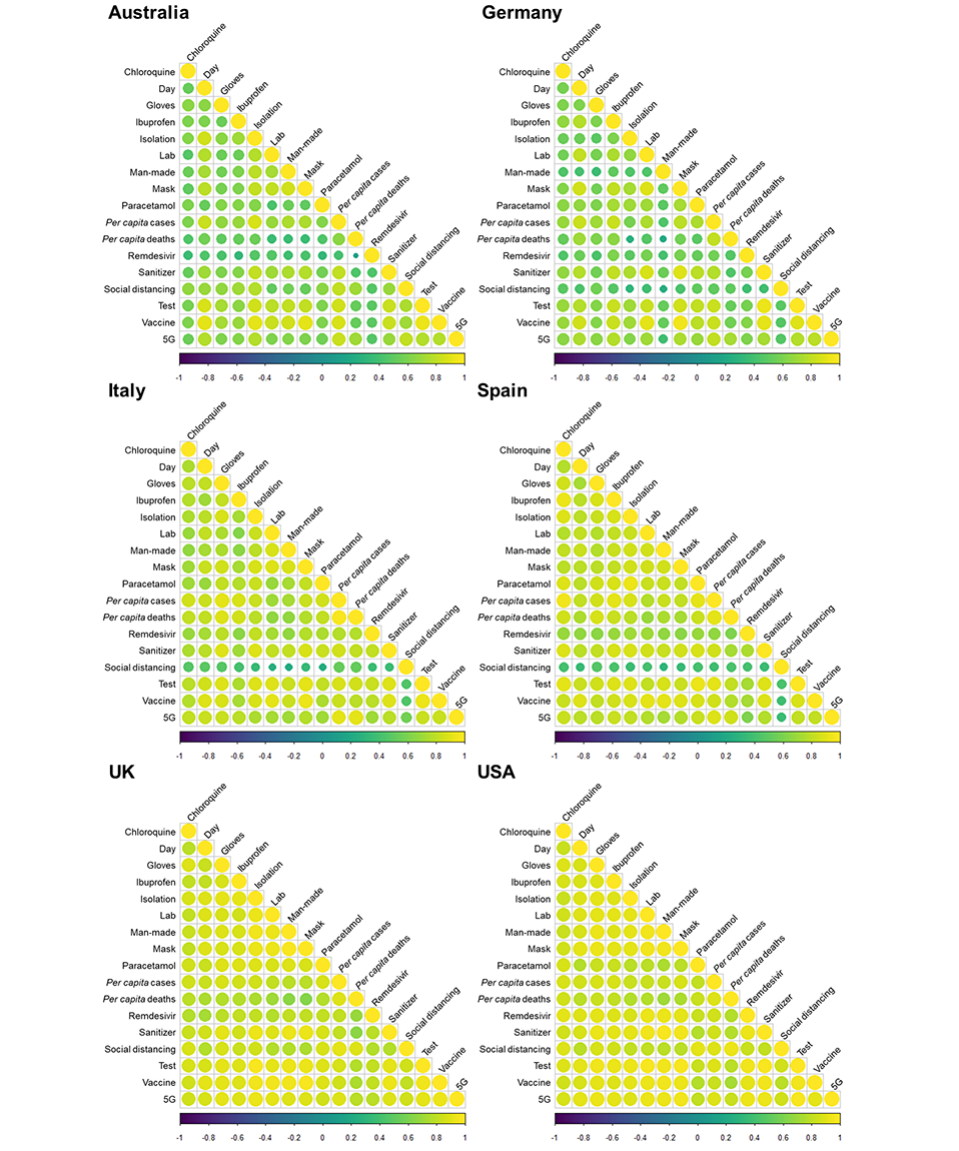
Correlograms for the search factors and per capita case and death rates for each country. The size of each circle indicates the strength of the correlation as does the color, denoted by the scale bar, with yellow and purple denoting positive and negative correlations, respectively.

In all countries, almost all normalized search terms significantly positively correlated with one another (overall mean Spearman rho 0.753, SD 0.158) and *per capita* deaths (mean *per capita* deaths Spearman rho 0.690, SD 0.168) and cases (mean *per capita* cases Spearman rho 0.800, SD 0.112; Figure 8, Table 1, and Multimedia Appendix 1 Table S1); the only exception was the nonsignificant association between *per capita* deaths and remdesivir RSV in Australia (Spearman rho 0.134, *P*=.08). Overall, stronger correlations were identified more universally for the United Kingdom (mean Spearman rho 0.851, SD 0.066) and the United States (mean Spearman rho 0.873, SD 0.058), while relatively weaker correlations were shown for Australia (mean Spearman rho 0.641, SD 0.150) and Germany (mean Spearman rho 0.632, SD 0.157; Figure 8, Table 1, and Multimedia Appendix 1 Table S1). In Italy and Spain, the weakest correlations were those between social distancing and all other variables.

**Table 1:** Mean Spearman rho rank coefficients and their standard deviations are given for each country and overall results for all six countries.

Countries	Overall correlation, mean (SD)	Time correlation, mean (SD)	Cases correlation, mean (SD)	Deaths correlation, mean (SD)
All	0.753 (0.158)	0.769 (0.122)	0.800 (0.112)	0.690 (0.168)
Australia	0.641 (0.150)	0.701 (0.136)	0.732 (0.122)	0.495 (0.146)
Germany	0.632 (0.157)	0.681 (0.143)	0.719 (0.123)	0.535 (0.153)
Italy	0.753 (0.147)	0.772 (0.093)	0.819 (0.103)	0.796 (0.104)
Spain	0.766 (0.152)	0.764 (0.120)	0.826 (0.133)	0.762 (0.116)
UK	0.851 (0.066)	0.835 (0.052)	0.846 (0.037)	0.750 (0.075)
US	0.873 (0.058)	0.861 (0.047)	0.858 (0.024)	0.802 (0.056)

## Discussion

This study aims to identify any correlation between the internet searching of defined COVID-19–relevant search terms and the *per capita* cases and deaths in six countries. We identified a positive correlation between the cases and deaths relating to COVID-19 and online searches surrounding government policies, medical interventions, and scientific misinformation.

### Principal Results

Between November 1, 2019 and April 17, 2020, *per capita* deaths and cases showed a similar trend across the six countries, with all having reached or passed peak daily new cases during the first wave of the pandemic. However, Australia and Germany experienced fewer deaths during this time period, allowing for a direct comparison of the search trends across countries with high and low COVID-19 cases and deaths. Where the ratio of mortality to cases is higher, such as the United Kingdom, which had the highest excess deaths in Europe during this period [35], this could reflect strained health care provision, delayed or reduced effectiveness of preventative measures, a poorer testing effort, or a combination of all of these [36,37]. Disparity in testing across countries may also have exacerbated differences in mortality. The importance of testing is illustrated by its high RSV across all countries (Figure 5) and the finding that the greatest degree of testing (Germany) aligns with relatively low mortality and weak correlations between RSVs and caseload. Where testing and contact tracing have been employed (eg, Germany, South Korea), they have been undoubtedly effective in mitigating increases in cases and deaths [38,39], possibly leading to an increased media and public interest, predominantly, it seems, in countries where it is lacking.

Overall, stronger correlations were observed in the United Kingdom and the United States. The English-speaking majority of these countries could explain this, given the widespread use of English on social media and in international news. In direct contrast, Australia had some of the weakest overall correlations; the combined low *per capita* deaths and cases, and the earlier application of travel restrictions and a 2 week quarantine [40,41] may have fostered a greater sense of safety and, therefore, less need by individuals to focus on the pandemic, evidenced by reduced interest in medical interventions. The overall strength of correlations being weakest in Australia and Germany, where the case and death figures are lower, supports the association between reduced public pressure and a less coordinated uptake of news and misinformation.

That, in almost all cases, *per capita* deaths and cases correlated with the search term RSVs further suggests a strong relationship between the pressure elicited upon the public and their receptibility to pandemic-related digital information. The virus was internationally recognized and regularly reported by January [2], with many of the proposed preventative measures and medical interventions being widely searched online before cases and deaths began to emerge (Figures 5 and 6). The peak of most RSVs in mid-March, aligning approximately with peak *per capita* deaths and cases (Figures 1 and 2), also coincides with the beginning of lockdown in many countries [42], suggesting that populations were well informed prelockdown and ready for substantial changes to living conditions. The more dramatic peaks of search term RSVs following the beginning of March may denote the public searching news-relevant topics in far greater volume due to their willingness to follow government guidance, increased anxiety, and free time. In Italy, however, RSV peaks arrived earlier, likely due to the earlier arrival of the virus. The later peaks, which are often larger, may be propagated by greater exposure to mainstream and social media while at home and increased levels of anxiety (Multimedia Appendix 1 Figure S1) [43,44] thus creating a “second wave.” This relatively erratic persistent search behavior, particularly surrounding misinformation, could indicate heightened public panic especially as per capita deaths increase.

The data in this study highlights the utility of infoveillance in assessing public readiness for and adoption of preventative measures. The early interest in masks observed in the United States and Australia could indicate a willingness for, or pre-emptive fear of, the use of PPE. Despite some antimask sentiment in politicians [45] and possible reluctance by governments to impose mask-wearing for fear of appearing dictatorial, the public may be more prepared for discourse surrounding PPE than expected given the high RSVs. Conversely, social distancing consistently correlated weakly with other search terms, specifically in Germany, Italy, and Spain, despite all three countries entering nationwide lockdowns and observing government-mandated social distancing rules. Given the use of translated search terms, where these received more searches than the English equivalent, this is unlikely to be due to linguistic differences, despite these comprising only the non–English-speaking countries. In some countries, strict enforcement of social distancing may not have been necessary due to greater compliance with guidelines (Germany). Alternatively, social distancing may not have been so heavily emphasized or adhered to in some countries, resulting in government-enforced curfews with fines for noncompliance, as experienced in Italy [46,47]. Regardless, clear and repeated guidance should be provided by governments to ensure compliance by their citizens. Good government response has been credited with the rapid reduction of lockdown measures in some countries, but such responses need to be data driven [38,48], and GT can provide an effective proxy for the extent of public adherence to this guidance.

Public interest in medical interventions was similarly moderately consistent between countries, with ibuprofen and chloroquine being the most searched. Some of this search intensity likely arose from misinformation, for example, the high RSVs for ibuprofen coincided with a scientific correspondence to The Lancet hypothesizing a heightened risk to a subset of patients with hypertension and diabetes should they take ibuprofen to combat COVID-19 [21]. This correspondence became misrepresented on messaging platforms and in media [16,49] as “evidence” that ibuprofen worsened COVID-19 symptoms. Furthermore, a second ibuprofen RSV peak in April in the United States coincided with a viral social media message claiming that patients with COVID-19 using ibuprofen did not recover [16]. The European Medicines Agency and the US Food and Drug Administration quickly discredited this as misinformation, possibly explaining the ephemerality of the RSV peak [25,26]. Paracetamol was highly searched simultaneously with ibuprofen, suggesting that people were seeking alternatives [50]. That the ibuprofen RSV comprises the highest medical intervention search peak in the United Kingdom and Germany, and a relatively high peak in other countries, compared to lower RSVs for experimental COVID-19 disease-modifying drugs such as remdesivir [51], confirms the capacity of misinformation to penetrate the public consciousness. Although this may also reflect the less familiar names and scientific background of the experimental drugs. This is further evidenced by the much larger RSVs for *vaccine* across all countries, a term familiar with most people, yet a therapeutic option that is clearly much further from public availability than therapies such as remdesivir [52]. The second peak of interest in vaccines in the United Kingdom was likely propagated by UK media reporting the initiation of clinical trials at the University of Oxford [53]. It is worth noting, however, that one experimental drug, namely, chloroquine, was searched with far greater intensity in the United States. This is likely due to US government briefings that supported chloroquine as a potential treatment for COVID-19 [54] based on a small clinical study [55], which led to multiple larger studies that ultimately did not support the outcomes [55,56] with most trials now suspended as reviewed in [57] and following some reports of accidental self-poisoning [58]. The important role of clear guidance from government is further exemplified from the suggestion during US government briefings that consideration should be given to the internal use of disinfectant and UV light in combating COVID-19. This is a clear example of misinformation arising from misinterpreted scientific literature that led to widescale panic and increased calls to poison centers [59-61].

Similarly, mass media and elected representatives have also propagated theories that SARS-CoV-2 is either man-made or was leaked from a laboratory in Wuhan. A quickly retracted scientific preprint appeared to propagate this theory by providing it an undue sense of credibility [24]. Although the man-made theory was scientifically discredited [27], public discussion moved toward a “leak” of the virus [16,23], highlighting the evolution and adaptability of misinformation, especially when supported by public figures [62]. Editors, reviewers, and authors should maintain stringent safeguards to ensure appropriate publishing, even of preprints, especially regarding such sensitive topics [63]. Similar conspiracy theories with large RSVs (Figure 7) arose via mainstream and social media outlets suggesting the spread of COVID-19 by 5G towers. The theory itself was in early circulation and despite being discredited as misinformation in January, long before the search intensity peaked [22], it led to vigilante attacks on phone masts and engineers in uninformed attempts to arrest viral spread [3,16,22]. The danger of misguided intervention led by misinformation outlines a clear requirement for mechanisms to reduce the spread of, while rationally and widely discrediting, these theories via perceivably credible sources such as national governments or professional medical bodies [1]. That the search volume surrounding 5G and ibuprofen dissipated so rapidly after documented attempts made by public health authorities such as the World Health Organization to curb the spread of this misinformation [25,26] best illustrates this point. It is, therefore, clear that during this pandemic the consumption of mass media, social media, government announcements, and health organization releases has influenced the public perception around both the causes and treatments of COVID-19 and, as perhaps best evidenced by the high RSVs for ibuprofen and 5G, has contributed to both public panic and health care issues such as reduced stocks of essential medicines caused by stockpiling [50].

### Limitations of GT Data

This study used GT data for six countries in which it is the most popular, but not the only, internet search engine. However, as the most widely used, it provides the best snapshot of user searches so that appropriate statistical studies can be conducted. As with any searches, the data presented in this study do not confirm subscription of those searching the terms to the ideals, interventions, or policies that they represent; many of the queries that contribute to these data may have been submitted by critics and sceptics. Even such searches, however, ratify the increased public awareness, discussion, and spread of the information denoted by the search terms. A greater volume of people reached by the information will undoubtedly suggest a greater number subscribing to the theories and ideas. The progression of a global pandemic is incredibly complicated and unpredictable, and the findings of this study focus on GT data from just one time period in a currently ongoing situation. Although this study bears relevance primarily to the beginning of the pandemic, this is arguably the most critical point at which to limit spread; however, the findings may not prove as relevant to periods when the public have adjusted to the situation.

### Conclusions

Infoveillance has already proven to be a valuable tool during the COVID-19 pandemic through detection of novel symptoms [11], assessment of behaviors such as self-medication [64], and identification of outbreaks [65]. This study focuses on the public response during the early developing pandemic, particularly surrounding misinformation, government policy, and medical interventions.

A study exploring the use of GT for digital epidemiology found that search term RSVs were influenced far more by media clamor than by epidemiological burden [66]. This pandemic is unique in that rapidly emerging medical research deposited in preprint archives has been accessible and consumed by the media and public pre–peer review, leading to potentially dangerous misinterpretation, as has occurred with chloroquine [58]. Although our findings ratify this, we also identified a positive correlation between internet searching and COVID-19 deaths and cases, indicating a more synergistic combined effect of epidemiological burden and media attention. The prevalence and online spread of misinformation has been reported previously for COVID-19 [1] with regard to social media platforms, and, as in this study, the findings ultimately identified an important role for public health organizations and governments in providing accessible online information and refutation of misinformation. Medical misinformation has drastic health care consequences and pre-existing misinformation, particularly that surrounding vaccines, will be a significant future obstacle in overcoming COVID-19 [6]. The presentation of accurate information, including infodemiology data as illustrated in this study, to maintain societal ease is vital, and there is an imperative for scientists, public health authorities, and governments to collaborate to rigorously maintain this.

## Appendix

Multimedia Appendix 1 Table S1 and Figure S1.

## Abbreviations

BBSRC: Biotechnology and Biological Sciences Research Council
GT: Google Trends
PPE: personal protective equipment
RSV: relative search volume
