# Supplementary material for: Investigating the Prevalence of Reactive Online Searching in the COVID-19 Pandemic: Infoveillance Study
Source: J Med Internet Res. 2020 Oct 27;22(10):e19791. doi: 10.2196/19791 (PMC7595752; doi:10.2196/19791)
Supplement: Multimedia Appendix 1 [file jmir_v22i10e19791_app1.docx]

**Table S1. Correlation values for search term RSVs, *per capita* cases and deaths, and progressive days.**

| **Factor 1** | **Factor 2** | **Australia Correlation** | **Australia *P*-value** | **Germany Correlation** | **Germany *P*-value** | **Italy Correlation** | **Italy *P*-value** | **Spain Correlation** | **Spain *P*-value** | **UK Correlation** | **UK *P*-value** | **USA Correlation** | **USA *P*-value** |
| --- | --- | --- | --- | --- | --- | --- | --- | --- | --- | --- | --- | --- | --- |
| Day | 5G | 0.771 | <.001 | 0.753 | <.001 | 0.822 | <.001 | 0.793 | <.001 | 0.875 | <.001 | 0.913 | <.001 |
| Day | Chloroquine | 0.521 | <.001 | 0.581 | <.001 | 0.747 | <.001 | 0.779 | <.001 | 0.797 | <.001 | 0.836 | <.001 |
| 5G | Chloroquine | 0.552 | <.001 | 0.656 | <.001 | 0.806 | <.001 | 0.826 | <.001 | 0.84 | <.001 | 0.872 | <.001 |
| Day | Gloves | 0.645 | <.001 | 0.607 | <.001 | 0.815 | <.001 | 0.732 | <.001 | 0.834 | <.001 | 0.877 | <.001 |
| 5G | Gloves | 0.604 | <.001 | 0.66 | <.001 | 0.83 | <.001 | 0.792 | <.001 | 0.902 | <.001 | 0.927 | <.001 |
| Chloroquine | Gloves | 0.659 | <.001 | 0.62 | <.001 | 0.781 | <.001 | 0.853 | <.001 | 0.889 | <.001 | 0.885 | <.001 |
| Day | Ibuprofen | 0.6 | <.001 | 0.776 | <.001 | 0.716 | <.001 | 0.792 | <.001 | 0.816 | <.001 | 0.875 | <.001 |
| 5G | Ibuprofen | 0.623 | <.001 | 0.766 | <.001 | 0.792 | <.001 | 0.824 | <.001 | 0.88 | <.001 | 0.909 | <.001 |
| Chloroquine | Ibuprofen | 0.618 | <.001 | 0.679 | <.001 | 0.765 | <.001 | 0.928 | <.001 | 0.806 | <.001 | 0.877 | <.001 |
| Gloves | Ibuprofen | 0.558 | <.001 | 0.617 | <.001 | 0.803 | <.001 | 0.872 | <.001 | 0.869 | <.001 | 0.89 | <.001 |
| Day | Isolation | 0.842 | <.001 | 0.554 | <.001 | 0.794 | <.001 | 0.795 | <.001 | 0.881 | <.001 | 0.882 | <.001 |
| 5G | Isolation | 0.77 | <.001 | 0.62 | <.001 | 0.754 | <.001 | 0.815 | <.001 | 0.926 | <.001 | 0.894 | <.001 |
| Chloroquine | Isolation | 0.623 | <.001 | 0.552 | <.001 | 0.759 | <.001 | 0.884 | <.001 | 0.869 | <.001 | 0.833 | <.001 |
| Gloves | Isolation | 0.652 | <.001 | 0.483 | <.001 | 0.875 | <.001 | 0.828 | <.001 | 0.917 | <.001 | 0.909 | <.001 |
| Ibuprofen | Isolation | 0.709 | <.001 | 0.613 | <.001 | 0.728 | <.001 | 0.879 | <.001 | 0.91 | <.001 | 0.887 | <.001 |
| Day | Lab | 0.772 | <.001 | 0.804 | <.001 | 0.802 | <.001 | 0.813 | <.001 | 0.887 | <.001 | 0.897 | <.001 |
| 5G | Lab | 0.601 | <.001 | 0.704 | <.001 | 0.72 | <.001 | 0.767 | <.001 | 0.904 | <.001 | 0.932 | <.001 |
| Chloroquine | Lab | 0.466 | <.001 | 0.562 | <.001 | 0.683 | <.001 | 0.769 | <.001 | 0.818 | <.001 | 0.854 | <.001 |
| Gloves | Lab | 0.558 | <.001 | 0.582 | <.001 | 0.755 | <.001 | 0.768 | <.001 | 0.868 | <.001 | 0.912 | <.001 |
| Ibuprofen | Lab | 0.543 | <.001 | 0.746 | <.001 | 0.629 | <.001 | 0.813 | <.001 | 0.844 | <.001 | 0.9 | <.001 |
| Isolation | Lab | 0.77 | <.001 | 0.678 | <.001 | 0.831 | <.001 | 0.836 | <.001 | 0.921 | <.001 | 0.904 | <.001 |
| Day | Man-made | 0.739 | <.001 | 0.409 | <.001 | 0.723 | <.001 | 0.828 | <.001 | 0.864 | <.001 | 0.874 | <.001 |
| 5G | Man-made | 0.63 | <.001 | 0.382 | <.001 | 0.702 | <.001 | 0.726 | <.001 | 0.884 | <.001 | 0.925 | <.001 |
| Chloroquine | Man-made | 0.558 | <.001 | 0.459 | <.001 | 0.667 | <.001 | 0.816 | <.001 | 0.831 | <.001 | 0.835 | <.001 |
| Gloves | Man-made | 0.565 | <.001 | 0.37 | <.001 | 0.745 | <.001 | 0.772 | <.001 | 0.882 | <.001 | 0.921 | <.001 |
| Ibuprofen | Man-made | 0.618 | <.001 | 0.511 | <.001 | 0.648 | <.001 | 0.836 | <.001 | 0.873 | <.001 | 0.887 | <.001 |
| Isolation | Man-made | 0.792 | <.001 | 0.449 | <.001 | 0.797 | <.001 | 0.841 | <.001 | 0.941 | <.001 | 0.905 | <.001 |
| Lab | Man-made | 0.724 | <.001 | 0.452 | <.001 | 0.868 | <.001 | 0.848 | <.001 | 0.924 | <.001 | 0.951 | <.001 |
| Day | Mask | 0.788 | <.001 | 0.846 | <.001 | 0.824 | <.001 | 0.793 | <.001 | 0.881 | <.001 | 0.906 | <.001 |
| 5G | Mask | 0.609 | <.001 | 0.755 | <.001 | 0.774 | <.001 | 0.775 | <.001 | 0.895 | <.001 | 0.954 | <.001 |
| Chloroquine | Mask | 0.519 | <.001 | 0.628 | <.001 | 0.716 | <.001 | 0.798 | <.001 | 0.819 | <.001 | 0.845 | <.001 |
| Gloves | Mask | 0.585 | <.001 | 0.563 | <.001 | 0.849 | <.001 | 0.787 | <.001 | 0.875 | <.001 | 0.931 | <.001 |
| Ibuprofen | Mask | 0.605 | <.001 | 0.802 | <.001 | 0.724 | <.001 | 0.811 | <.001 | 0.872 | <.001 | 0.911 | <.001 |
| Isolation | Mask | 0.818 | <.001 | 0.726 | <.001 | 0.877 | <.001 | 0.844 | <.001 | 0.936 | <.001 | 0.908 | <.001 |
| Lab | Mask | 0.809 | <.001 | 0.89 | <.001 | 0.897 | <.001 | 0.847 | <.001 | 0.939 | <.001 | 0.956 | <.001 |
| Man-made | Mask | 0.849 | <.001 | 0.513 | <.001 | 0.863 | <.001 | 0.848 | <.001 | 0.961 | <.001 | 0.957 | <.001 |
| Day | Paracetamol | 0.524 | <.001 | 0.667 | <.001 | 0.69 | <.001 | 0.795 | <.001 | 0.822 | <.001 | 0.761 | <.001 |
| 5G | Paracetamol | 0.578 | <.001 | 0.688 | <.001 | 0.72 | <.001 | 0.83 | <.001 | 0.858 | <.001 | 0.815 | <.001 |
| Chloroquine | Paracetamol | 0.61 | <.001 | 0.649 | <.001 | 0.72 | <.001 | 0.894 | <.001 | 0.859 | <.001 | 0.866 | <.001 |
| Gloves | Paracetamol | 0.63 | <.001 | 0.603 | <.001 | 0.812 | <.001 | 0.841 | <.001 | 0.889 | <.001 | 0.838 | <.001 |
| Ibuprofen | Paracetamol | 0.63 | <.001 | 0.732 | <.001 | 0.777 | <.001 | 0.873 | <.001 | 0.896 | <.001 | 0.821 | <.001 |
| Isolation | Paracetamol | 0.636 | <.001 | 0.7 | <.001 | 0.828 | <.001 | 0.928 | <.001 | 0.883 | <.001 | 0.785 | <.001 |
| Lab | Paracetamol | 0.434 | <.001 | 0.707 | <.001 | 0.699 | <.001 | 0.826 | <.001 | 0.861 | <.001 | 0.787 | <.001 |
| Man-made | Paracetamol | 0.551 | <.001 | 0.482 | <.001 | 0.728 | <.001 | 0.836 | <.001 | 0.883 | <.001 | 0.754 | <.001 |
| Mask | Paracetamol | 0.51 | <.001 | 0.736 | <.001 | 0.808 | <.001 | 0.818 | <.001 | 0.866 | <.001 | 0.784 | <.001 |
| Day | Remdesivir | 0.454 | <.001 | 0.548 | <.001 | 0.736 | <.001 | 0.67 | <.001 | 0.737 | <.001 | 0.881 | <.001 |
| 5G | Remdesivir | 0.473 | <.001 | 0.619 | <.001 | 0.7 | <.001 | 0.602 | <.001 | 0.78 | <.001 | 0.92 | <.001 |
| Chloroquine | Remdesivir | 0.375 | <.001 | 0.448 | <.001 | 0.687 | <.001 | 0.698 | <.001 | 0.773 | <.001 | 0.901 | <.001 |
| Gloves | Remdesivir | 0.429 | <.001 | 0.431 | <.001 | 0.79 | <.001 | 0.654 | <.001 | 0.795 | <.001 | 0.917 | <.001 |
| Ibuprofen | Remdesivir | 0.328 | <.001 | 0.61 | <.001 | 0.651 | <.001 | 0.695 | <.001 | 0.802 | <.001 | 0.882 | <.001 |
| Isolation | Remdesivir | 0.476 | <.001 | 0.492 | <.001 | 0.813 | <.001 | 0.705 | <.001 | 0.819 | <.001 | 0.876 | <.001 |
| Lab | Remdesivir | 0.474 | <.001 | 0.593 | <.001 | 0.722 | <.001 | 0.704 | <.001 | 0.779 | <.001 | 0.927 | <.001 |
| Man-made | Remdesivir | 0.383 | <.001 | 0.4 | <.001 | 0.677 | <.001 | 0.676 | <.001 | 0.804 | <.001 | 0.928 | <.001 |
| Mask | Remdesivir | 0.425 | <.001 | 0.599 | <.001 | 0.778 | <.001 | 0.659 | <.001 | 0.812 | <.001 | 0.916 | <.001 |
| Paracetamol | Remdesivir | 0.323 | <.001 | 0.533 | <.001 | 0.756 | <.001 | 0.686 | <.001 | 0.805 | <.001 | 0.83 | <.001 |
| Day | Sanitizer | 0.709 | <.001 | 0.761 | <.001 | 0.8 | <.001 | 0.796 | <.001 | 0.842 | <.001 | 0.876 | <.001 |
| 5G | Sanitizer | 0.697 | <.001 | 0.734 | <.001 | 0.78 | <.001 | 0.778 | <.001 | 0.883 | <.001 | 0.93 | <.001 |
| Chloroquine | Sanitizer | 0.534 | <.001 | 0.663 | <.001 | 0.756 | <.001 | 0.891 | <.001 | 0.838 | <.001 | 0.852 | <.001 |
| Gloves | Sanitizer | 0.601 | <.001 | 0.578 | <.001 | 0.889 | <.001 | 0.808 | <.001 | 0.914 | <.001 | 0.928 | <.001 |
| Ibuprofen | Sanitizer | 0.622 | <.001 | 0.806 | <.001 | 0.713 | <.001 | 0.875 | <.001 | 0.844 | <.001 | 0.904 | <.001 |
| Isolation | Sanitizer | 0.809 | <.001 | 0.747 | <.001 | 0.892 | <.001 | 0.871 | <.001 | 0.932 | <.001 | 0.915 | <.001 |
| Lab | Sanitizer | 0.714 | <.001 | 0.87 | <.001 | 0.805 | <.001 | 0.854 | <.001 | 0.891 | <.001 | 0.945 | <.001 |
| Man-made | Sanitizer | 0.745 | <.001 | 0.553 | <.001 | 0.808 | <.001 | 0.834 | <.001 | 0.937 | <.001 | 0.966 | <.001 |
| Mask | Sanitizer | 0.776 | <.001 | 0.888 | <.001 | 0.874 | <.001 | 0.847 | <.001 | 0.935 | <.001 | 0.943 | <.001 |
| Paracetamol | Sanitizer | 0.576 | <.001 | 0.79 | <.001 | 0.827 | <.001 | 0.871 | <.001 | 0.868 | <.001 | 0.779 | <.001 |
| Remdesivir | Sanitizer | 0.446 | <.001 | 0.589 | <.001 | 0.801 | <.001 | 0.741 | <.001 | 0.797 | <.001 | 0.922 | <.001 |
| Day | Social distancing | 0.687 | <.001 | 0.436 | <.001 | 0.489 | <.001 | 0.352 | <.001 | 0.774 | <.001 | 0.786 | <.001 |
| 5G | Social distancing | 0.804 | <.001 | 0.472 | <.001 | 0.56 | <.001 | 0.373 | <.001 | 0.841 | <.001 | 0.844 | <.001 |
| Chloroquine | Social distancing | 0.678 | <.001 | 0.493 | <.001 | 0.474 | <.001 | 0.413 | <.001 | 0.878 | <.001 | 0.896 | <.001 |
| Gloves | Social distancing | 0.679 | <.001 | 0.579 | <.001 | 0.475 | <.001 | 0.469 | <.001 | 0.863 | <.001 | 0.869 | <.001 |
| Ibuprofen | Social distancing | 0.715 | <.001 | 0.473 | <.001 | 0.414 | <.001 | 0.427 | <.001 | 0.822 | <.001 | 0.844 | <.001 |
| Isolation | Social distancing | 0.766 | <.001 | 0.317 | <.001 | 0.356 | <.001 | 0.377 | <.001 | 0.846 | <.001 | 0.817 | <.001 |
| Lab | Social distancing | 0.503 | <.001 | 0.397 | <.001 | 0.261 | <.001 | 0.406 | <.001 | 0.779 | <.001 | 0.807 | <.001 |
| Man-made | Social distancing | 0.561 | <.001 | 0.259 | <.001 | 0.24 | .002 | 0.311 | <.001 | 0.765 | <.001 | 0.783 | <.001 |
| Mask | Social distancing | 0.537 | <.001 | 0.43 | <.001 | 0.353 | <.001 | 0.353 | <.001 | 0.752 | <.001 | 0.799 | <.001 |
| Paracetamol | Social distancing | 0.656 | <.001 | 0.51 | <.001 | 0.247 | 0.001 | 0.419 | <.001 | 0.877 | <.001 | 0.919 | <.001 |
| Remdesivir | Social distancing | 0.474 | <.001 | 0.392 | <.001 | 0.361 | <.001 | 0.406 | <.001 | 0.774 | <.001 | 0.835 | <.001 |
| Sanitizer | Social distancing | 0.686 | <.001 | 0.384 | <.001 | 0.362 | <.001 | 0.375 | <.001 | 0.775 | <.001 | 0.82 | <.001 |
| Day | Test | 0.864 | <.001 | 0.764 | <.001 | 0.856 | <.001 | 0.822 | <.001 | 0.872 | <.001 | 0.913 | <.001 |
| 5G | Test | 0.776 | <.001 | 0.769 | <.001 | 0.83 | <.001 | 0.8 | <.001 | 0.917 | <.001 | 0.963 | <.001 |
| Chloroquine | Test | 0.581 | <.001 | 0.699 | <.001 | 0.789 | <.001 | 0.91 | <.001 | 0.874 | <.001 | 0.888 | <.001 |
| Gloves | Test | 0.72 | <.001 | 0.624 | <.001 | 0.891 | <.001 | 0.856 | <.001 | 0.909 | <.001 | 0.949 | <.001 |
| Ibuprofen | Test | 0.658 | <.001 | 0.824 | <.001 | 0.747 | <.001 | 0.885 | <.001 | 0.896 | <.001 | 0.93 | <.001 |
| Isolation | Test | 0.858 | <.001 | 0.719 | <.001 | 0.897 | <.001 | 0.898 | <.001 | 0.968 | <.001 | 0.932 | <.001 |
| Lab | Test | 0.785 | <.001 | 0.827 | <.001 | 0.893 | <.001 | 0.808 | <.001 | 0.902 | <.001 | 0.961 | <.001 |
| Man-made | Test | 0.81 | <.001 | 0.522 | <.001 | 0.838 | <.001 | 0.861 | <.001 | 0.927 | <.001 | 0.959 | <.001 |
| Mask | Test | 0.809 | <.001 | 0.858 | <.001 | 0.918 | <.001 | 0.826 | <.001 | 0.921 | <.001 | 0.963 | <.001 |
| Paracetamol | Test | 0.614 | <.001 | 0.819 | <.001 | 0.811 | <.001 | 0.923 | <.001 | 0.886 | <.001 | 0.829 | <.001 |
| Remdesivir | Test | 0.484 | <.001 | 0.595 | <.001 | 0.835 | <.001 | 0.731 | <.001 | 0.808 | <.001 | 0.946 | <.001 |
| Sanitizer | Test | 0.831 | <.001 | 0.92 | <.001 | 0.889 | <.001 | 0.891 | <.001 | 0.909 | <.001 | 0.968 | <.001 |
| Social distancing | Test | 0.741 | <.001 | 0.494 | <.001 | 0.445 | <.001 | 0.426 | <.001 | 0.85 | <.001 | 0.849 | <.001 |
| Day | Vaccine | 0.906 | <.001 | 0.858 | <.001 | 0.894 | <.001 | 0.898 | <.001 | 0.913 | <.001 | 0.874 | <.001 |
| 5G | Vaccine | 0.762 | <.001 | 0.781 | <.001 | 0.837 | <.001 | 0.816 | <.001 | 0.921 | <.001 | 0.92 | <.001 |
| Chloroquine | Vaccine | 0.618 | <.001 | 0.686 | <.001 | 0.764 | <.001 | 0.835 | <.001 | 0.845 | <.001 | 0.843 | <.001 |
| Gloves | Vaccine | 0.729 | <.001 | 0.629 | <.001 | 0.875 | <.001 | 0.802 | <.001 | 0.905 | <.001 | 0.908 | <.001 |
| Ibuprofen | Vaccine | 0.681 | <.001 | 0.838 | <.001 | 0.722 | <.001 | 0.86 | <.001 | 0.898 | <.001 | 0.895 | <.001 |
| Isolation | Vaccine | 0.907 | <.001 | 0.715 | <.001 | 0.877 | <.001 | 0.878 | <.001 | 0.95 | <.001 | 0.903 | <.001 |
| Lab | Vaccine | 0.839 | <.001 | 0.913 | <.001 | 0.903 | <.001 | 0.911 | <.001 | 0.947 | <.001 | 0.935 | <.001 |
| Man-made | Vaccine | 0.848 | <.001 | 0.533 | <.001 | 0.83 | <.001 | 0.908 | <.001 | 0.956 | <.001 | 0.943 | <.001 |
| Mask | Vaccine | 0.919 | <.001 | 0.951 | <.001 | 0.927 | <.001 | 0.885 | <.001 | 0.96 | <.001 | 0.935 | <.001 |
| Paracetamol | Vaccine | 0.61 | <.001 | 0.793 | <.001 | 0.747 | <.001 | 0.878 | <.001 | 0.894 | <.001 | 0.777 | <.001 |
| Remdesivir | Vaccine | 0.486 | <.001 | 0.621 | <.001 | 0.784 | <.001 | 0.72 | <.001 | 0.815 | <.001 | 0.913 | <.001 |
| Sanitizer | Vaccine | 0.823 | <.001 | 0.916 | <.001 | 0.886 | <.001 | 0.885 | <.001 | 0.923 | <.001 | 0.946 | <.001 |
| Social distancing | Vaccine | 0.718 | <.001 | 0.475 | <.001 | 0.455 | <.001 | 0.385 | <.001 | 0.817 | <.001 | 0.799 | <.001 |
| Test | Vaccine | 0.929 | <.001 | 0.896 | <.001 | 0.925 | <.001 | 0.878 | <.001 | 0.942 | <.001 | 0.952 | <.001 |
| Day | Per capita cases | 0.821 | <.001 | 0.826 | <.001 | 0.825 | <.001 | 0.819 | <.001 | 0.822 | <.001 | 0.845 | <.001 |
| 5G | Per capita cases | 0.828 | <.001 | 0.813 | <.001 | 0.901 | <.001 | 0.884 | <.001 | 0.889 | <.001 | 0.893 | <.001 |
| Chloroquine | Per capita cases | 0.605 | <.001 | 0.696 | <.001 | 0.844 | <.001 | 0.948 | <.001 | 0.853 | <.001 | 0.867 | <.001 |
| Gloves | Per capita cases | 0.67 | <.001 | 0.702 | <.001 | 0.911 | <.001 | 0.859 | <.001 | 0.879 | <.001 | 0.876 | <.001 |
| Ibuprofen | Per capita cases | 0.693 | <.001 | 0.832 | <.001 | 0.866 | <.001 | 0.917 | <.001 | 0.855 | <.001 | 0.86 | <.001 |
| Isolation | Per capita cases | 0.866 | <.001 | 0.595 | <.001 | 0.821 | <.001 | 0.874 | <.001 | 0.85 | <.001 | 0.839 | <.001 |
| Lab | Per capita cases | 0.685 | <.001 | 0.751 | <.001 | 0.712 | <.001 | 0.767 | <.001 | 0.812 | <.001 | 0.84 | <.001 |
| Man-made | Per capita cases | 0.758 | <.001 | 0.449 | <.001 | 0.728 | <.001 | 0.825 | <.001 | 0.787 | <.001 | 0.827 | <.001 |
| Mask | Per capita cases | 0.745 | <.001 | 0.785 | <.001 | 0.828 | <.001 | 0.811 | <.001 | 0.791 | <.001 | 0.864 | <.001 |
| Paracetamol | Per capita cases | 0.631 | <.001 | 0.753 | <.001 | 0.843 | <.001 | 0.915 | <.001 | 0.865 | <.001 | 0.812 | <.001 |
| Remdesivir | Per capita cases | 0.408 | <.001 | 0.561 | <.001 | 0.764 | <.001 | 0.679 | <.001 | 0.802 | <.001 | 0.858 | <.001 |
| Sanitizer | Per capita cases | 0.817 | <.001 | 0.769 | <.001 | 0.853 | <.001 | 0.855 | <.001 | 0.815 | <.001 | 0.854 | <.001 |
| Social distancing | Per capita cases | 0.815 | <.001 | 0.519 | <.001 | 0.52 | <.001 | 0.393 | <.001 | 0.91 | <.001 | 0.882 | <.001 |
| Test | Per capita cases | 0.841 | <.001 | 0.823 | <.001 | 0.872 | <.001 | 0.905 | <.001 | 0.861 | <.001 | 0.881 | <.001 |
| Vaccine | Per capita cases | 0.867 | <.001 | 0.824 | <.001 | 0.837 | <.001 | 0.85 | <.001 | 0.853 | <.001 | 0.831 | <.001 |
| Day | Per capita deaths | 0.58 | <.001 | 0.703 | <.001 | 0.818 | <.001 | 0.752 | <.001 | 0.742 | <.001 | 0.785 | <.001 |
| 5G | Per capita deaths | 0.719 | <.001 | 0.727 | <.001 | 0.906 | <.001 | 0.854 | <.001 | 0.8 | <.001 | 0.825 | <.001 |
| Chloroquine | Per capita deaths | 0.448 | <.001 | 0.523 | <.001 | 0.818 | <.001 | 0.85 | <.001 | 0.796 | <.001 | 0.857 | <.001 |
| Gloves | Per capita deaths | 0.501 | <.001 | 0.602 | <.001 | 0.881 | <.001 | 0.869 | <.001 | 0.775 | <.001 | 0.825 | <.001 |
| Ibuprofen | Per capita deaths | 0.535 | <.001 | 0.641 | <.001 | 0.857 | <.001 | 0.833 | <.001 | 0.766 | <.001 | 0.803 | <.001 |
| Isolation | Per capita deaths | 0.559 | <.001 | 0.27 | <.001 | 0.793 | <.001 | 0.797 | <.001 | 0.758 | <.001 | 0.769 | <.001 |
| Lab | Per capita deaths | 0.377 | <.001 | 0.478 | <.001 | 0.662 | <.001 | 0.691 | <.001 | 0.7 | <.001 | 0.761 | <.001 |
| Man-made | Per capita deaths | 0.368 | <.001 | 0.214 | .005 | 0.668 | <.001 | 0.742 | <.001 | 0.649 | <.001 | 0.718 | <.001 |
| Mask | Per capita deaths | 0.372 | <.001 | 0.562 | <.001 | 0.78 | <.001 | 0.727 | <.001 | 0.639 | <.001 | 0.756 | <.001 |
| Paracetamol | Per capita deaths | 0.448 | <.001 | 0.494 | <.001 | 0.812 | <.001 | 0.821 | <.001 | 0.775 | <.001 | 0.835 | <.001 |
| Remdesivir | Per capita deaths | 0.134 | .081 | 0.464 | <.001 | 0.763 | <.001 | 0.608 | <.001 | 0.681 | <.001 | 0.773 | <.001 |
| Sanitizer | Per capita deaths | 0.451 | <.001 | 0.446 | <.001 | 0.812 | <.001 | 0.737 | <.001 | 0.645 | <.001 | 0.773 | <.001 |
| Social distancing | Per capita deaths | 0.706 | <.001 | 0.511 | <.001 | 0.539 | <.001 | 0.429 | <.001 | 0.886 | <.001 | 0.926 | <.001 |
| Test | Per capita deaths | 0.536 | <.001 | 0.537 | <.001 | 0.847 | <.001 | 0.807 | <.001 | 0.76 | <.001 | 0.798 | <.001 |
| Vaccine | Per capita deaths | 0.532 | <.001 | 0.574 | <.001 | 0.8 | <.001 | 0.766 | <.001 | 0.745 | <.001 | 0.738 | <.001 |
| Per capita cases | Per capita deaths | 0.657 | <.001 | 0.807 | <.001 | 0.978 | <.001 | 0.908 | <.001 | 0.885 | <.001 | 0.894 | <.001 |


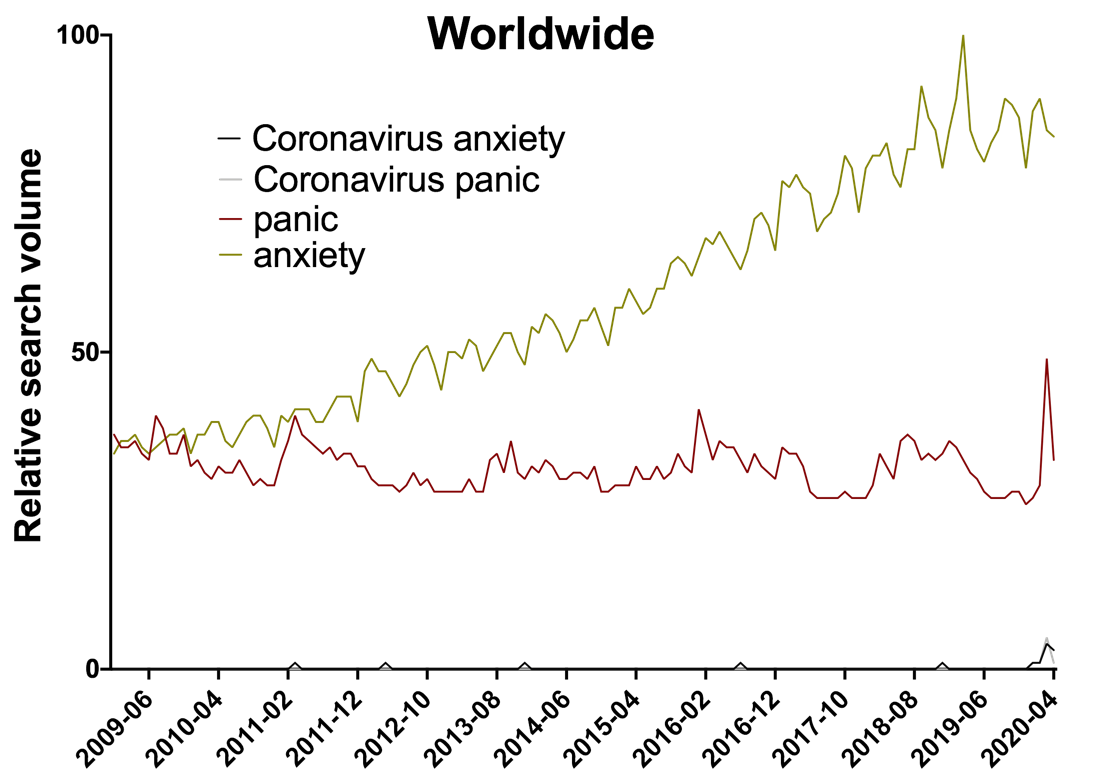


**Figure S1. Relative search intensity for “coronavirus anxiety”, “coronavirus panic”, “panic” and “anxiety” during and preceding the COVID-19 pandemic.**
